# Risk Adapted Management of Febrile Neutrepenia and Early Cessation of Empirical Antibiotherapy in Hematopoietic Stem Cell Transplantation Setting

**DOI:** 10.4274/balkanmedj.2016.0012

**Published:** 2017-03-28

**Authors:** Ali Hakan Kaya, Emre Tekgündüz, Fazilet Duygu, Dicle Koca, Filiz Bekdemir, Hikmetullah Batgi, Bahar Ulu Uncu, Tuğçe Nur Yiğenoğlu, Mehmet Sinan Dal, Merih Çakar Kızıl, Fevzi Altuntaş

**Affiliations:** 1 Hematology and Bone Marrow Transplant Unit, Dr. Abdurrahman Yurtaslan Oncology Training and Research Hospital, Ankara, Turkey; 2 Clinic of Infectious Diseases, Dr. Abdurrahman Yurtaslan Oncology Training and Research Hospital, Ankara, Turkey

**Keywords:** Haematopoietic stem cell transplantation, Febrile neutropenia, early cessation of empirical antibiotherapy

## Abstract

**Background:**

Haematopoietic stem cell transplantation is a curative treatment option for many haematological disorders. Infection following haematopoietic stem cell transplantation is one of the major causes of mortality.

**Aims:**

To investigate the outcomes of early cessation of empirical antibiotic treatment per protocol in febrile neutropenia patients who have undergone haematopoietic stem cell transplantation at our clinic.

**Study Design:**

Descriptive study.

**Methods:**

The present study retrospectively evaluated febrile neutropenia attacks in haematopoietic stem cell transplantation recipients during the period June 2014 - January 2015 at our haematopoietic stem cell transplantation clinic.

**Results:**

A total of 72 febrile neutropenia attacks were evaluated in 53 patients. In 46 febrile neutropenia attacks, microbiologic cultures revealed positive results. In culture-positive febrile neutropenia episodes a single bacterium was isolated in 32 cases and multiple strains were isolated in 14. In 15 patients, empirical antibiotic therapy was discontinued after 72 hours. These patients were clinically stable, without evident focus of infection and had negative culture results. Only 4 recurrent episodes were observed (27%) after cessation of antibiotherapy. No patient died as a result of recurrent infection. The 30-day and 100-day post-transplantation mortality rates of patients with febrile neutropenia episodes were 11.3% (6/53) and 3.8% (2/53), respectively. Infection-related 30-day and 100-day mortality rates were 7.5% (4/53) and 0% (0/53), respectively.

**Conclusion:**

The main message of our study is that early cessation of empirical antibiotherapy seems to be feasible in eligible patients without increasing febrile neutropenia mortality rates.

Haematopoietic stem cell transplantation (HSCT) is a curative treatment option for several potentially lethal conditions including haematological cancers and bone marrow failure. HSCT is being increasingly utilized globally, including in elderly patients, due to improvements in the conditioning regimen and supportive care. Infections are among the leading cause of morbidity and mortality in patients who have undergone HSCT. HSCT recipients are highly susceptible to infections due to the primary disease itself requiring HSCT and as a result of iatrogenic immunosuppression induced by conditioning regimens and prophylaxis/treatment of graft versus-host disease (GVHD) following HSCT (1,2).

Prompt recognition and treatment of febrile neutropenia (FN) is crucial to prevent the development of sepsis and subsequent mortality (3,4). The Infectious Diseases Society of America (IDSA) and European Conference on Infections in Leukaemia (ECIL) have published guidelines for the treatment of FN, which are accepted on a global scale. However, the local antibiotic resistance profile, as well as dominant local pathogens must be taken into account when initiating empirical antibiotic treatment (5).

The present study aimed to investigate the outcomes of early cessation of empirical antibiotic treatment per protocol in febrile neutropenic patients who have undergone HSCT at our clinic.

## MATERIAL AND METHODS

The present study retrospectively evaluated FN attacks in HSCT recipients during the period of June 2014 - January 2015 at our HSCT clinic.

Previously defined criteria are used to diagnose FN episodes (3). The risk category of patients was defined by the Multinational Association for Supportive Care in Cancer score (6). The European Organization for the Research and Treatment of Cancer/Mycoses Study Group (EORCT/MSG) criteria were used to define invasive fungal infections (IFI) in the study cohort (7). A new FN episode was considered when fever and neutropenia re-occurred in a patient who remained afebrile for at least 72 hours after discontinuation of antibiotics.

### Assessment

History and detailed physical examination were carried out during the initial assessment of patients assumed to have FN. For patients who had fever and/or signs of infection the following tests were performed: chest X-ray, urinalysis, cultures of peripheral blood, catheter (central or peripheral) and urine. Additional investigations were done as clinically indicated. Blood cultures were taken in the first 3 days in four sets. In patients with ongoing fever, two sets of blood cultures were obtained on the first day of FN attack. On the second and third days of the episode only one set of blood culture was evaluated daily. Samples were taken from both catheter lumens and peripheral vein in patients who had central venous catheters (CVCs), and from both arms in patients who had no CVCs. No further blood cultures were taken from patients whose fever subsided. No new blood cultures were obtained in patients who completed four sets.

### Prophylaxis

After they entered the neutropenic period, all allo-HSCT patients were treated with fluconazole (Zolax; Sanovel, Sarıyer, İstanbul, Turkey) 400 mg/day until day +75, valacyclovir (Valtrex; Glaxo Smith Kline Turkey, Levent, İstanbul, Turkey) 1000 mg/day until day +90 and trimethoprim (TMP) - sulfamethoxazole (SMX) (Bactrim Fort; Deva, Küçükçekmece, İstanbul, Turkey) (320 mg/week TMP equivalent) until day +180. In auto-HSCT, prophylaxis regimen included fluconazole 200 mg/day, valacyclovir 1000 mg/day and TMP/SMX 320 mg/week, from the time they entered the neutropenic period until engraftment.

### Treatment

**Figure 1:**
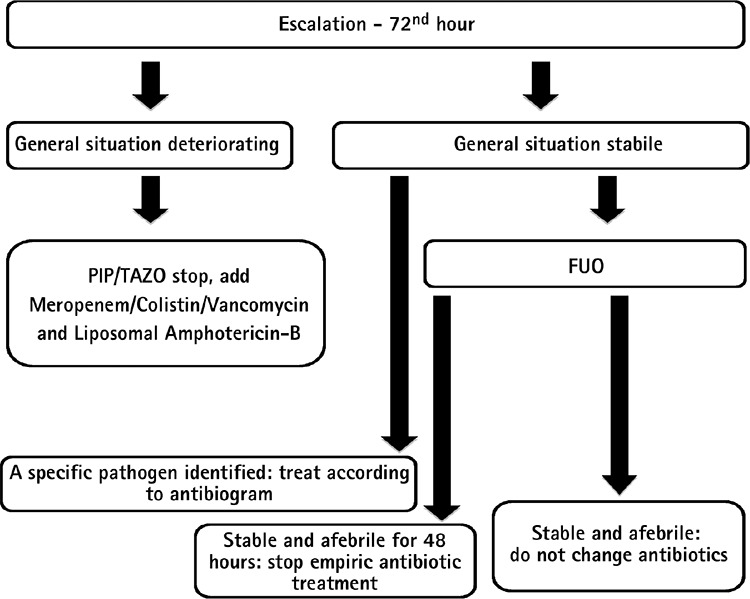
Patient follow-up in the escalation strategy. *FUO: Fever of unknown origin, PIP/TAZO: Piperacillin tazobactam*

**Figure 2:**
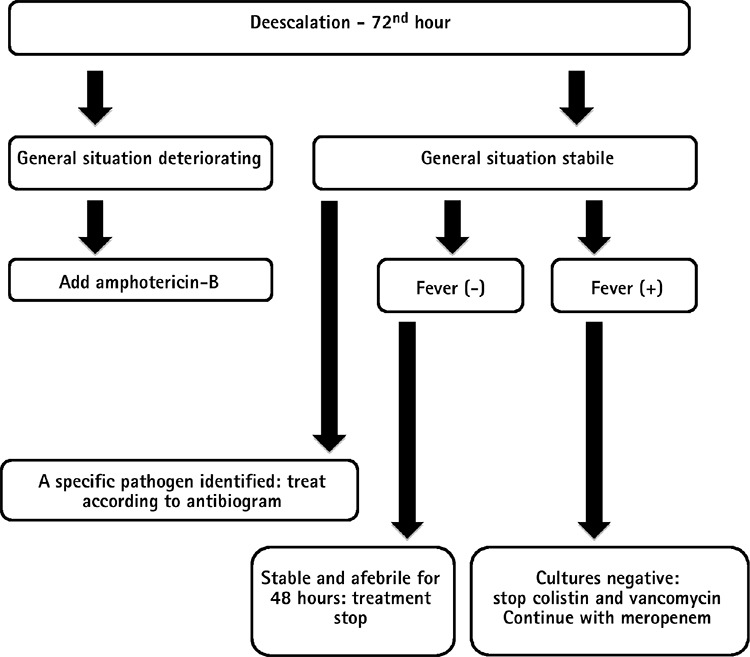
Patient follow-up in the de-escalation strategy.

In the empiric approach, escalation or de-escalation strategies were applied to patients in line with the ECIL-4 guideline. While an empiric ultra-broad-spectrum therapy (de-escalation) was administered to patients who had a high risk of mortality during assessment, the first-line treatment was a narrower spectrum therapy (escalation) in patients with a relatively lower risk of mortality. The de-escalation criteria are depicted in Table 1. The patients who did not satisfy the de-escalation criteria were treated within the framework of the escalation approach. Piperacillin/tazobactam (PIP/TAZO) (Tazocin; Phizer Turkey, Ortaköy, İstanbul, Turkey) 18 gm/day was started for the escalation group and meropenem (Meronem; Astra Zeneca Turkey, Levent, İstanbul, Turkey) 3 gm/day, colistin (Colimycin; Koçak Farma, Bağcılar, İstanbul, Turkey) 5 mg/kg/day and vancomycin (Vancotek; Koçak Farma, Bağcılar, İstanbul, Turkey) 2 gm/day 4 for the de-escalation group. The empiric vancomycin therapy was administered to patients who were applied the escalation approach only when they met the certain criteria mentioned in the IDSA-2010 guideline (Table 2). TMP/SMX 15 mg/kg/day 4 (TMP equivalent) was started for patients who developed hypoxaemia with suspected *P. jirovecii* pneumonia during their follow-up. The patients who did not respond to 3 days of broad-spectrum antibiotherapy were assessed with computed tomography (CT) of the thorax for signs of occult IFI. If the conditions necessitated a de-escalation in patients who had started escalation therapy, a transition was made according to the de-escalation protocol without waiting for the outcome of the 3 days of empiric antibiotic therapy. After the 72^nd^ hour of the first-line empiric therapy was completed, the further pathways chosen in the escalation and de-escalation group of patients are summarized in Figure 1 and Figure 2.

**Figure 3:**
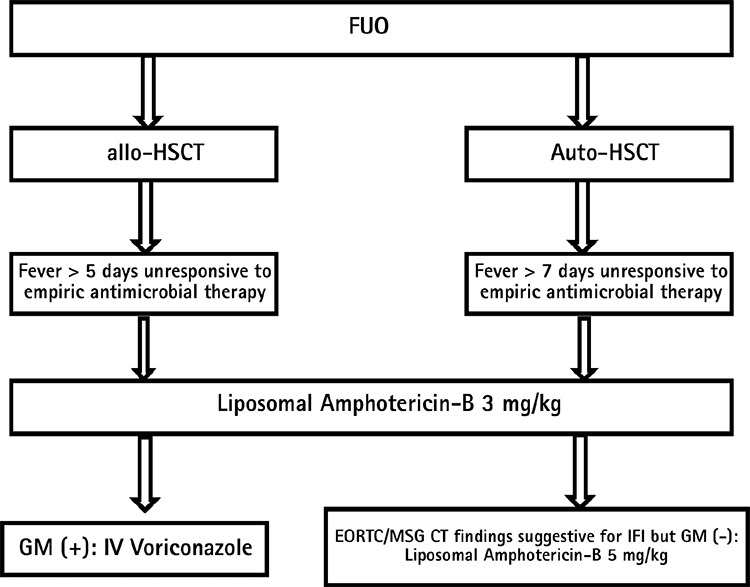
Empiric approach to IFI. *IFI: invasive fungal infections; FUO: fever of unknown origin; allo-HSCT: allogeneic hematopoietic stem cell transplantation; auto-HSCT: autologous hematopoietic stem cell transplantation; GM: galatmannan; IV: intravenous; EORT/MSG: European Organization fort he Research and Treatment of Cancer/Mycoses Study Group; CT: computerized tomography*

All patients were checked for serum galactomannan (GM) and cytomegalovirus (CMV) polimerase chain reaction (PCR) twice a week during the time they were hospitalized. A serum GM value ≥0.5 and CMV copy counts >500/mL with quantitative PCR in two successive blood samples are considered significant levels for the triggering of empiric and/or pre-emptive therapy. The standard pre-emptive approach was used against CMV infection and an empiric therapy against IFIs. The patients were assessed in accordance with the current guidelines for IFI development (8). Briefly, those who were administered auto-HSCT were considered to be in the low-risk group and those administered allo-HSCT in the high-risk group. After assessing the clinical and laboratory findings of patients, 4 liposomal amphotericin-B (Ambisome; Gilead Turkey, Beşiktaş, İstanbul, Turkey) 3 mg/kg/day was started when no response was obtained to the empiric antibiotherapy for 7 days in the auto-HSCT group and for 5 days in the allo-HSCT group. The patients were assessed with their thorax CT and serum GM results. If the clinical signs contained in the EORTC/MSG guideline (nodule±halo sign, air crescent sign, cavitation, etc.) were detected in a thoracic CT, the amphotericin-B dose was increased to 5 mg/kg/day. In the case of GM positivity in two successive serum, sputum and/or bronchoalveolar lavage samples, amphotericin-B was stopped and 4 voriconazole (Vfend; Phizer Turkey, Ortaköy, İstanbul, Turkey) therapy was started. The empiric antifungal therapy was administered for 14 days in patients who did not have any findings of fungal infection other than isolated fever and whose general conditions did not worsen (Figure 3).

### Statistical analyses

The study was designed as a retrospective cohort analysis. Descriptive statistics (number, percentage, median) were made as indicated. All statistical analyses were performed with IBM SPSS Statistics for Windows, Version 21.0. (IBM Corp: Armonk, New York, USA) software. This study was approved by the local Ethics Committee and written informed consent was obtained from the study participants.

## RESULTS

Demographic and clinical features of patients are summarized in Table 3. A total of 72 FN attacks were evaluated in 53 patients. In 46 FN attacks, microbiologic cultures revealed positive results. In culture-positive FN episodes a single bacterium was isolated in 32 cases and multiple strains were isolated in 14. The foci of infection in culture-positive FN episodes are depicted in Table 4. In culture-negative FN episodes (n=26), a focus for infection was evident in 11 episodes. In five of these episodes the focus for infection was lung and in the remaining six the focus was identified as soft tissue. Gram (-) bacteria were isolated in 30 and gram (+) bacteria were isolated in 10 FN episodes. In six episodes both gram (-) and gram (+) strains were isolated (Table 5).

The frequency of microbiological drug resistance in gram (-) bacteria was as follows: PIP/TAZO 33% (n=16); fluoroquinolones 25% (n=12); amikacin 10% (n=5); and carbapenems 4% (n=2). Seventy-five per cent (n=15), 10% (n=2), 10% (n=2) and 5% (n=1) gram (+) organisms were resistant to fluoroquinolones, vancomycin, teicoplanin and ampicillin, respectively.

Empirical antifungal therapy was given in 13 episodes of FN, which were observed in 11 patients. In three of these patients there was possible or probable fungal infection according to EORCT/MSG criteria. 

In 15 patients, empirical antibiotic therapy was discontinued after 72 hours. These patients were clinically stable, without evident focus of infection and had negative culture results. Only four recurrent episodes were observed (27%) after cessation of antibiotherapy. No patient died as a result of recurrent infection.

The 30-day and 100-day post-transplantation mortality rates of patients with FN episodes were 11.3% (6/53) and 15.1% (8/53), respectively. Infection-related mortality at days 30 and 100 was the same as 7.5% (4/53). No infection-related mortality was observed during the 30-100-day period.

## DISCUSSION

The main message of our study is that early cessation of empirical antibiotherapy seems to be feasible in eligible patients without increasing FN mortality rates. As suggested in the ECIL-4 guideline, empirical antibiotic treatment can be discontinued after 72 hours in haemodynamically stable, culture-negative patients without evident focus of infection, who remain afebrile for at least 48 hours. The empirical antibiotic treatment was discontinued in our study in patients who met the above criteria. Despite having a limited number of patients, we think that early antibiotic discontinuation in patients meeting the above criteria is a reasonable and safe practice considering the fact that the recurrence rate of FN episodes was 27% (4/15) with no infection-related mortality. The ECIL-4 recommendation seems to be advantageous in preventing antibiotic resistance and reducing costs as compared to the conservative approach, where the empiric antibiotherapy is continued until resolution of fever and neutropenia. Still, it should be noted that there is no consensus among experts on this subject.

According to the recent Centre for International Blood and Marrow Transplant Research data, infection is the third leading cause of death in patients undergoing HSCT, after GVHD and recurrence of the primary disease (9). Prompt administration of empiric broad-spectrum antibiotics is therefore crucial in patients with FN (4). Bacterial infections are the major cause of mortality in immunocompromised patients, followed by fungal infections (10,11). One of the controversial issues is fluoroquinolone prophylaxis in patients who have high risk for development of FN. Such prophylaxis is recommended in IDSA-2010 and ECIL-4 guidelines. Bucaneve et al. (12), in their placebo-controlled study, found that levofloxacin prophylaxis resulted in significantly reduced bouts of fever as well as decreased rates of infections caused by gram (-) bacilli in high-risk neutropenic patients. In a meta-analysis on the subject (13), levofloxacin prophylaxis in high-risk cancer patients was found to be associated with increased survival. IDSA recommends fluroquinolone prophylaxis in patients who have undergone allo-HSCT. Patients who will receive auto-HSCT, however, seem to possess a low risk of serious bacterial infection even in the case of prolonged neutropenia (>7 days). For this reason, fluroquinolone prophylaxis is not generally recommended in such patients (3).

Cattaneo et al. (14) have observed an increase in gram (-) infection rates in patients who received fluroquinolone prophylaxis. The authors suggested that the increased fluroquinolone resistance in E. coli strains is the reason for this unexpected result. It has been recently shown that the rate of resistance to levofloxacin in Turkey has been around 60-80%, which is also in agreement with our local data (15,16). In the present cohort, fluroquinolone prophylaxis was not given due to a high local resistance pattern.

Two patients in escalation and de-escalation groups were lost due to infection. Our results showed that despite the ultra-broad-spectrum antibiotherapy involving meropenem, colistin and vancomycin, the infection-related death rate was very high in the de-escalation group (50%). On the other hand, the mortality rate in patients who underwent escalation was 4.1%. The results suggest that risk stratification according to ECIL-4 (escalation/de-escalation) is quite reasonable in terms of predicting outcome. Additionally, this approach seems to prevent the use of some unnecessary drugs with potential side effects and is cost-effective.

Studies report that bloodstream infections are generally seen in 15-25% of FN episodes, while in 20-40% of febrile neutropenic patients the focus of infection was lungs. On the other hand, in 10-40% of patients the source of infection/fever remains undetermined (3,10,17). Approximately 67% of the microorganisms identified in our study involved the bloodstream or lungs. The cause of fever could not be determined in 20.8% of the FN attacks. In the light of our data and above-mentioned studies, the blood-circulatory system and lungs should be considered the most frequent focuses of infection in patients presenting with FN.

Looking at the literature, gram (-) and gram (+) agents are seen at rates of between 60-80% and 20-40% in febrile neutropenic patients, respectively (18,19,20,21). In the present study, we identified gram (-) microorganisms in 30 patients (65%), while in 10 (22%) and 6 (13%) patients, gram (+) and polymicrobial agents were isolated, respectively. The most frequently isolated gram (-) agents were *E. coli, Klebsiella* and *Pseudomonas* (20,21,22). The results of HITIT-2 (23), a multicentre gram (-) surveillance study made in Turkey in 2007, also showed that the most frequently found agents were *E. coli* and *K. pneumonia*. Again according to HITIT-2 data, the high ESBL positivity rate in *E. coli* strains and increased fluoroquinolone resistance were underlined and the PIP/TAZO resistance in *E. coli* and *Klebsiella* were found to be 18% and 25.4%, respectively. Similar rates of PIP/TAZO resistance were also observed in our study (*E. coli*: 20% and *Klebsiella*: 13%). In gram (-) strains the PIP/TAZO and carbapenem resistance rates were 33% and 4%, respectively. Due to high PIP/TAZO resistance in our clinic, we decided to revise our FN protocol. In the resistance profile of gram (+) agents in our study, we thought that the resistances to vancomycin, teicoplanin and ampiciline were within acceptable limits except for that of fluoroquinolon (75%).

According to the guidelines and studies, the risk of developing IFI is 15-25% in allo-HSCT and 2-, 6% in auto-HSCT (3,18,23). Basically two types of approaches can be chosen in the management of IFIs: pre-emptive or empirical (3,7,18). According to the pivotal study conducted by Maertens et al. (24), a pre-emptive approach based on daily GM monitoring and early thoracic CT examinations, decreases antifungal use by 78% (antifungal use being 35.5% in the empirical group and 7.7% in the pre-emptive group). Which of the pre-emptive and empirical approaches should be preferred in preventing invasive fungal infections is controversial (25). For a pre-emptive procedure, fast and effective working microbiology laboratories and imaging facilities will obviously be mandatory. We thought that an empirical approach would be more appropriate at our site because of logistics. The rate of using antifungal drugs in our study was 21% for all patients. Liposomal amphotericin-B was preferred in the empirical approach as it was fungicidal to both yeasts and moulds.

Even though there have been significant developments made in diagnostic modalities and treatment strategies with respect to FN in recent years, the mortality rate is still between 5 and 15% (26). The studies carried out in our country report that the FN-related mortality rate ranges between 12 and 31% in patients with haematological malignancy (27,28,29). The 100-day infection-related mortality rate in our study (7.5%) seems to be in line with previous experience worldwide.

FN in HSCT is a condition that requires an urgent multidisciplinary treatment approach. Each haematology clinic should be able to formulate its own treatment model, which requires keeping regular records of local microbiological trends. Implementation of a treatment strategy based on their local data may contribute to increased overall survival through reduction of infection-related mortality rates in HSCT. Our results indicate that in eligible patients, empirical antibiotherapy can be stopped in the early time period in patients with FN according to written local protocol without causing harm. This strategy should be ideally confirmed in appropriately designed randomized trials in order to draw firm conclusions.

## Figures and Tables

**Table 1:**
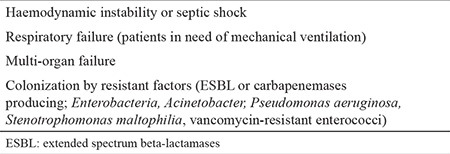
Criteria for de-escalation

**Table 2:**
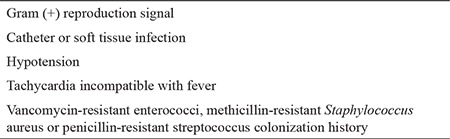
Indications for vancomycin in patients receiving escalation therapy

**Table 3:**
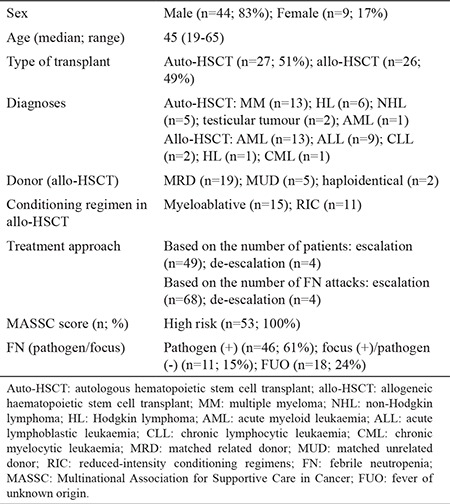
Demographic and clinical characteristics of patients

**Table 4:**
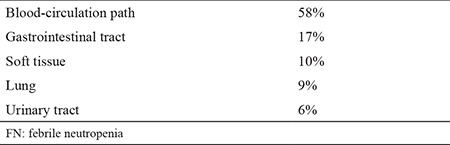
Distribution of focuses of infection in FN episodes

**Table 5:**
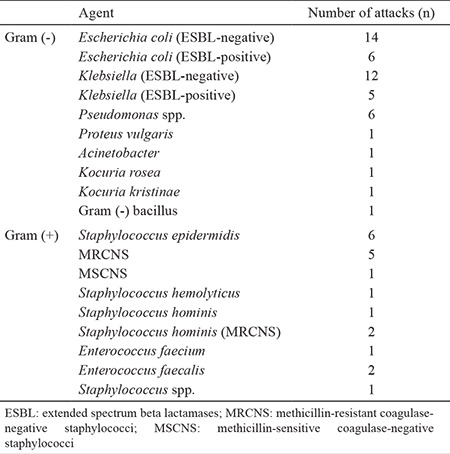
Distribution of isolated pathogens

## References

[1] (2014). Pasquini MC, Zhu X. Current use and outcome of hematopoietic stem cell transplantation: CIBMTR summary slides.

[2] Basak GW, Wiktor-Jedrzejczak W, Labopin M, Schoemans H, Ljungman P, Kobbe G, et al (2015). Allogeneic hematopoietic stem cell transplantation in solid organ transplant recipients: a retrospective, multicenter study of the EBMT. Am J Transplant.

[3] Freifeld AG, Bow EJ, Sepkowitz KA, Boeckh MJ, Ito JI, Mullen CA, et al (2011). Clinical practice guideline for the use of antimicrobial agents in neutropenic patients with cancer: 2010 update by the infectious diseases Society of America. Clin Infect Dis.

[4] Zuckermann J, Moreira LB, Stoll P, Moreira LM, Kuchenbecker RS, Polanczyk CA (2008). Compliance with a critical pathway for the management of febrile neutropenia and impact on clinical outcomes. Ann Hematol.

[5] Sipsas NV, Bodey GP, Kontoyiannis DP (2005;15). Perspectives for the management of febrile neutropenic patients with cancer in the 21st century. Cancer.

[6] Uys A, Rapoport BL, Anderson R (2004). Febrile neutropenia: A prospective study to validate the Multinational Association of Supportive Care of Cancer (MASCC) risk-index score. Support Care Cancer.

[7] De Pauw B, Walsh TJ, Donnelly JP, Stevens DA, Edwards JE, Calandra T, et al (2008). Revised definitions of invasive fungal disease from the European Organization for Research and Treatment of Cancer/Invasive Fungal Infections Cooperative Group and the National Institute of Allergy and Infectious Diseases Mycoses Study Group (EORTC/MSG) Consensus Group. Clin Infect Dis.

[8] Barberan J, Mensa J, Llamas JC, Ramos IJ, Ruiz JC, Marín JR, et al (2011). Recommendations for the treatment of invasive fungal infection caused by filamentous fungi in the hematological patient. Rev Esp Quimioter.

[9] http://www.cibmtr.org.

[10] Sharma A, Lokeshwar N (2005). Febrile neutropenia in haematological malignancies. J Postgrad Med.

[11] Toussaint E, Bahel-Ball E, Vekemans M, Georgala A, Al-Hakak L, Paesmans M, et al (2006). Causes of fever in cancer patients. Support Care Cancer.

[12] Bucaneve G, Micozzi A, Menichetti F, Martino P, Dionisi MS, Martinelli G, et al (2005). Levofloxacin to prevent bacterial infection in patients with cancer and neutropenia. N Engl J Med.

[13] Gafter-Gvili A, Fraser A, Paul M, Leibovici L (2005). Meta-analysis: antibiotic prophylaxis reduces mortality in neutropenic patients. Ann Intern Med.

[14] Cattaneo C, Quaresmini G, Casari S, Capucci MA, Micheletti M, Borlenghi E, et al (2008). Recent changes in bacterial epidemiology and the emergence of fluoroquinolone-resistant Escherichia coli among patients with haematological malignancies: Results of a prospective study on 823 patients at a single institution. J Antimicrob Chemother.

[15] Erbay MÇ, Tekgündüz E, Çimentepe M, Akay N, İskender G, Tetik A, et al (2011). Kinolon resistance in patients with hematological malignancies.

[16] Köse Ş, Atalay S, Ödemiş İ, Adar P (2014). Çeşitli Klinik Örneklerden İzole Edilen Pseudomonas Aeruginosa Suşlarının Antibiyotik Duyarlılıkları. Ankem Derg.

[17] Akan H (2008). Febril Nötropeni. İç hastalıkları 1. baskı. Ed. Erol Ç. Ankara.

[18] Averbuch D, Orasch C, Cordonnier C, Livermore DM, Mikulska M, Viscoli C, et al (2013). European guidelines for empirical antibacterial therapy for febrile neutropenic patients in the era of growing resistance: summary of the 2011 4th. European Conference on Infections in Leukemia.

[19] Trecarichi EM, Tumbarello M, Spanu T, Caira M, Fianchi L, Chiusolo P, et al (2009). Incidence and clinical impact of extended-spectrum-beta-lactamase (ESBL) production and fluoroquinolone resistance in bloodstream infections caused by Escherichia coli in patients with hematological malignancies. J Infect.

[20] Sigurdardottir K, Digranes A, Harthug S, Nesthus I, Tangen JM, Dybdahl B, et al (2005). A multicentre prospective study of febrile neutropenia in Norway: microbiological fi ndings and antimicrobial susceptibility. Scand J Infect Dis.

[21] Demiraslan H, Yıldız O, Kaynar L, Altuntaş F, Eser B, Aygen B (2007). Febril nötropenik hastalardan izole edilen mikroorganizmalar ve antimikrobiyal duyarlılıkları: 2005 yılı verileri. Erciyes Tıp Derg.

[22] Gur D, Hascelik G, Aydin N, Telli M, Gültekin M, Ogülnç D, et al (2009). Antimicrobial resistance in Gram-negative hospital isolates: results of the Turkish HITIT-2 surveillance study of 2007. J Chemother.

[23] Marchetti O, Lamoth F, Mikulska M, Viscoli C, Verweij P, Bretagne S (2012). ECIL recommendations for the use of biological markers for the diagnosis of invasive fungal diseases in leukemic patients and hematopoietic SCT recipients. Bone Marrow Transplant.

[24] Maertens J, Theunissen K, Verhoef G, Verschakelen J, Lagrou K, Verbeken E, et al (2005). Galactomannan and Computed Tomography-Based Preemptive Antifungal Therapy in Neutropenic Patients at High Risk for Invasive Fungal Infection: A Prospective Feasibility Study. Clin Infect Dis.

[25] Cordonnier C, Pautas C, Maury S, Vekhoff A, Farhat H, Suarez F, et al (2009). Empirical versus preemptive antifungal therapy for high-risk, febrile, neutropenic patients: a randomized, controlled trial. Clin Infect Dis.

[26] Viscoli C, Varnier O, Machetti M (2005). Infections in patients with febrile neutropenia: Epidemiology, microbiology, and risk stratification. Clin Infect Dis.

[27] Sacar S, Hacioglu SK, Keskin A, Turgut H (2008). Evaluation of febrile neutropenic attacks in a tertiary care medical center in Turkey. J Infect Dev Ctries.

[28] Pehlivan M, Demirkan F, Özsan H, Yılmaz U, Ündar B (1999). Sitotoksik tedavi veya kemik iliği tutulumuna bağlı gelişen 148 febril nötropeni epizodu. Klimik Derg.

[29] Savaş L, Yıldırımak T, Önlen Y, Tan-Çetmeli G, Savaş N, Efe-İris N, ve ark (2006). Febril ve afebril hastalarda kan kültürlerinin değerlendirilmesi. Klimik Derg.

